# Hypofractionated intensity-modulated radiotherapy with concurrent chemotherapy for elderly patients with locally advanced pancreatic carcinoma

**DOI:** 10.1186/s13014-020-01712-2

**Published:** 2020-11-13

**Authors:** Takahiro Iwai, Michio Yoshimura, Ryo Ashida, Yoko Goto, Takahiro Kishi, Satoshi Itasaka, Keiko Shibuya, Masashi Kanai, Toshihiko Masui, Akihisa Fukuda, Hiroyoshi Isoda, Masahiro Hiraoka, Takashi Mizowaki

**Affiliations:** 1 Department of Radiation Oncology and Image-Applied Therapy, Graduate School of Medicine, Kyoto University, 54 Shogoin-Kawaharacho, Sakyo-ku, Kyoto, 606-8507 Japan; 2 Department of Radiation Oncology, Kurashiki Central Hospital, Okayama, Japan; 3 Department of Radiation Oncology, Graduate School of Medicine, Osaka City University, Osaka, Japan; 4 Department of Clinical Oncology, Kyoto University Graduate School of Medicine, Kyoto, Japan; 5 Department of Surgery, Kyoto University Graduate School of Medicine, Kyoto, Japan; 6 Department of Gastroenterology and Hepatology, Kyoto University Graduate School of Medicine, Kyoto, Japan; 7 Department of Diagnostic Imaging and Nuclear Medicine, Kyoto University Graduate School of Medicine, Kyoto, Japan; 8 Department of Radiation Oncology, Japanese Red Cross Society Wakayama Medical Center, Wakayama, Japan

## Abstract

**Background:**

It is important to understand how elderly patients with locally advanced pancreatic carcinoma (LAPC) should be treated, since the number of elderly cancer patients will increase. However, the optimal treatment for elderly patients with LAPC remains unclear. The purpose of this study was to evaluate the efficacy and safety of hypofractionated intensity-modulated radiotherapy (IMRT) with concurrent gemcitabine for elderly patients with LAPC.

**Methods:**

We retrospectively analysed the data from LAPC patients aged ≥ 75 years treated with hypofractionated IMRT (48 Gy in 15 fractions) with concurrent weekly gemcitabine at our institution from February 2013 to December 2018. Overall survival (OS), progression-free survival (PFS), locoregional progression-free survival (LRPFS), distant metastasis-free survival (DMFS), and the pattern of recurrence and toxicity were analysed.

**Results:**

Fifteen patients received treatment during the study period. The median age was 78 years (range 75–86 years), and the Eastern Cooperative Oncology Group (ECOG) performance status (PS) of all patients was 0–1. The median survival time (MST) and median PFS were 20.4 [95% confidence interval (CI) 10.3–36.8] and 13.5 (95% CI 6.4–20.3) months, respectively, and the 1-year OS and PFS rates were 80.0% (95% CI 50–93.1%) and 66.7% (95% CI 37.5–84.6%), respectively. The median LRPFS and median DMFS were 15.6 (95% CI 6.4–36.8) and 14.9 (95% CI 7.0–20.5) months, respectively, and the 1-year LRPFS and DMFS rates were 73.3% (95% CI 43.6–89.1%) and 66.7% (95% CI 37.5–84.6%), respectively. Non-haematologic grade 3 toxicity was observed in three cases, of which only one was induced by radiotherapy, whereas grade 4–5 non-haematologic acute or late toxicities were not observed.

**Conclusions:**

The OS and PFS of elderly patients with LAPC treated using hypofractionated IMRT with concurrent gemcitabine were favourable and without the occurrence of severe toxicity. This treatment strategy is feasible and promising for elderly LAPC patients with good PS.

## Background

Pancreatic carcinoma is a malignancy with a very poor prognosis. Approximately 30% of pancreatic carcinoma patients have unresectable locally advanced pancreatic carcinoma (LAPC) during diagnosis, with the prognosis of LAPC being poor [1]. The standard treatment for LAPC is chemotherapy (CTX) alone or concurrent chemoradiotherapy (CCRT). A previous study showed that CCRT was associated with decreasing local progression compared with CTX alone [2]. By contrast, the same trial described that the addition of radiotherapy (RT) to CTX did not improve overall survival (OS). However, an autopsy study showed that approximately 30% of patients with pancreatic cancer died because of locally destructive disease rather than distant metastasis [3]. Therefore, improving local control should be important for LAPC patients.

The treatment policy for LAPC remain controversial and the optimal treatment for elderly patients with LAPC is unclear. One of the reasons why the treatment for the elderly is still ill-defined is because the largest clinical trials have excluded elderly patients or have involved only a small number of them. For example, the trial that compared FOLFIRINOX with gemcitabine alone for metastatic pancreatic carcinoma registered patients aged 75 or younger [4], and the LAP07 trial that compared CCRT after induction CTX with CTX alone registered patients aged 71 or younger [2]. Therefore, it is meaningful to explore how elderly patients with LAPC should be treated, especially since the number of elderly patients with pancreatic cancer will increase within the next years [5].

Despite being promising for LAPC patients, the use of CCRT in pancreatic cancer could be problematic because of the anatomical relationship of the tumour with the surrounding organs. In particular, the gastrointestinal (GI) tracts such as the stomach or the duodenum are close to the pancreas, and excessive irradiation in these areas can cause radiation gastroenteritis, ulcer, perforation, or bleeding. Nevertheless, intensity-modulated radiotherapy (IMRT) can simultaneously reduce the dose to organ at risk (OAR), while assuring adequate target dose coverage compared to the conventional RT technique [6, 7]. Our previous research showed that the treatment using hypofractionated IMRT with full dose gemcitabine had improved OS and locoregional progression-free survival (LRPFS) without increasing GI toxicities, compared to the treatment using conventional RT with low-dose gemcitabine [8]. In the context of a poor prognosis disease, it is also important to shorten hospitalization, as long-term hospitalization has been shown to result in cognitive decline or disuse syndrome especially in elderly patients [9, 10]. For instance, hypofractionated RT is recommended for elderly patients with glioblastoma, a cancer that also has a very poor prognosis [11]. Hence, this treatment regimen of hypofractionated IMRT can be an ideal remedy for elderly patients with LAPC because of its low rate of GI toxicities and the short treatment period [12]. The purpose of this study was to investigate the efficacy and feasibility of hypofractionated IMRT with concurrent CTX for elderly patients with LAPC. To the best of our knowledge, this is the first study to evaluate the results of hypofractionated CCRT using IMRT for elderly LAPC patients.

## Methods

This was a retrospective study that reviewed data from all LAPC patients aged 75 or above who have been treated with definitive CCRT from February 2013 to December 2018 at our institution. Written informed consent was obtained from all the patients. This study was approved by the institutional Review Board of Kyoto University Hospital (R1048). The consensus regarding unresectable LAPC and the indication for definitive CCRT was assessed by the cancer board of our institution, which involves specialists of surgery, gastroenterology, medical oncology, diagnostic radiology, and radiation oncology. The indication for definitive CCRT was decided after checking whether the organ function or ECOG PS of the patient were within the tolerable limits for this treatment. Patients with gastrointestinal mucosa tumour invasion were excluded. The definition of unresectability was celiac axis or superior mesenteric artery > 180 degrees invasion, aortic invasion, or unreconstructible common hepatic artery or superior mesenteric vein, or portal occlusion [13]. Information regarding patient status, cancer stage, and treatment characteristics were obtained from the clinical records. The clinical stage was based on the 8th edition of the TNM classification for pancreas cancer of the Union for International Cancer Control (UICC) criteria [14]. Contrast-enhanced computed tomography (CT), dynamic magnetic resonance imaging (MRI) with Gadolinium-ethoxybenzyl-diethylenetriamine pentaacetic acid (Gd-EOB-DTPA), and ^18^F-flurodeoxyglucose-positron emission tomography (FDG-PET) imaging were performed to determine the clinical stage. For FDG-PET, the early examination was performed at 1 h post-injection, followed by delayed examination at 90–360 min post-injection if necessary. All patients were hospitalised during CCRT and were monitored for acute haematopoietic, GI, and other toxicities.

After CCRT, the patients were periodically followed up and evaluated through physical and blood examinations; CT scans of the chest-abdomen-pelvis were also obtained. The patients were also monitored for late GI toxicities. If they had symptoms of gastroduodenal ulcer or haemorrhage, such as dizziness or stomach aches, then oesophagogastroscopy was performed. Depending on the timing of the side effects, toxicities were classified as during induction CTX, acute (from the initiation of the treatment to 28 days after CCRT), and late toxicities. The Common Terminology Criteria for Adverse Events version 4.0 was used for the assessment of all toxicities [15].

### Chemotherapy

The treatment comprised induction CTX, CCRT, and maintenance CTX. The induction CTX regimen was based on gemcitabine (1000 mg/m^2^) that was administered intravenously once per week for 3 weeks, with 1-week rest. On the other hand, the CCRT regimen is comprised of RT, with a total dose of 48 Gy delivered in 15 fractions using IMRT, and weekly gemcitabine (1000 mg/m^2^). For maintenance CTX, weekly gemcitabine (1000 mg/m^2^) was administered for 3-weeks, with 1-week rest. Maintenance CTX was repeated until tumour progression, worsening of the patient’s condition, or the patient’s refusal. The gemcitabine dose was reduced, or dose interval was extended if the standard dose could not be administered because of toxicity. Depending on the patient’s medical condition, the regimen for induction and maintenance CTX were changed to gemcitabine in combination with nab-paclitaxel (125 mg/m^2^) for 3-weeks with 1-week rest at the discretion of the medical oncologist. When disease progression was detected, second-line CTX such as tegafur/gimeracil/oteracil potassium (S-1) or tegafur/uracil (UFT) was delivered if the patient’s condition was good enough to receive CTX [16, 17].

### Radiotherapy

For RT planning, 2-mm or 2.5-mm slice contrast-enhanced simulation CT was performed. To manage respiratory motion, breath-hold method, respiratory gating method, or dynamic tumour tracking method were adopted. The gross tumour volume (GTV) consisted of the primary tumour and metastatic lymph nodes. Contrast-enhanced CT was mainly used to determine the GTV, whereas MRI or FDG-PET were supplementally used. The clinical target volume (CTV) was defined as the GTV with a margin 5 mm in all directions plus the prophylactic area, which included the retropancreatic para-aortic lymph node and the neuroplexus involvement between the celiac axis and the superior mesenteric artery. The planning target volume (PTV) was CTV with a margin of 5 mm in all directions. The PTV-boost was the volume that subtracted the stomach plus 5 or 10 mm, and the duodenum plus 3- or 5-mm margins from the PTV. This margin was adjusted using the techniques for managing respiratory motion. The prescription dose was specified as D_95%_ (the dose that covers 95% of the structure) to PTV-boost = 48 Gy in 15 fractions and D_98%_ to PTV ≥ 36 Gy using simultaneous integrated boost (SIB)-IMRT technique. The dose constraints of OARs are listed in Table 1 [18]. If these dose constraints were not compatible with the dose prescriptions, dose prescriptions were decreased as D_50%_ to the PTV-boost = 48 Gy. All patients were treated five times per week with 6 MV or Flattening-Filter Free 10 MV photons on a linear accelerator, Truebeam™ (Varian Medical Systems, Inc, Palo Alto, California, USA) or Vero4DRT system (MHI-TM2000, Mitsubishi Heavy Industries, Ltd., Japan, and BrainLAB, Feldkirchen, Germany). Planning was performed with commercially available planning systems Eclipse™ (Varian, Medical Systems, Palo Alto, California, USA) or iPlan™ (Brainlab, Feldkirchen, Germany).

**Table 1:** Dose constraints for OAR

Structure	Constraints
Stomach/duodenum	V_45 Gy_ < 1 cc
	V_42 Gy_ < 5 cc
	V_39 Gy_ < 25 cc
Stomach + PRV/duodenum + PRV	V_39 Gy_ < 30 cc
	V_36 Gy_ < 45 cc
Spinal cord	D_max_ < 36 Gy
Spinal cord + PRV	D_2 cc_ < 39 Gy
Kidney (at least one)	V_20 Gy_ < 30%
Liver	D_mean_ < 30 Gy

OAR, organs at risk; PRV, planning organ at risk volume; D_max_, the maximum dose to the structure volume; D_mean_, the mean dose to the structure volume; D_2 cc_, the maximum dose covering ≥ 2 cc of the structure volume; V_xx Gy_, the volume of the structure receiving > xx Gy

### Statistics

OS was calculated from the starting date of induction CTX to the date of death by any cause and censored at the last follow-up visit for living patients. Progression-free survival (PFS), LRPFS, and distant metastasis-free survival (DMFS) were calculated from the starting date of induction CTX to disease progression or death; to locoregional disease progression or death; and to the detection of first distant metastasis or death, respectively. Disease progression was defined as a proof of progressive disease on CT or FDG-PET imaging using the Response Evaluation Criteria in Solid Tumors. Statistical analyses were performed using EZR version 1.41 (Saitama Medical Center, Jichi Medical University, Saitama, Japan). The Kaplan–Meier techniques were used to estimate the OS, PFS, LRPFS, and DMFS.

## Results

### Patient characteristics

Between February 2013 and December 2018, 18 LAPC patients aged 75 or above were eligible for definitive CCRT as per our cancer board. All patients underwent induction CTX for 1–6 months prior to RT, and 15 out of 18 patients received CCRT. The remaining three patients could not receive CCRT because of disease progression, refusal of chemoradiotherapy, and refusal of CTX. Fifteen patients undergoing CCRT were included in the analysis, patient and tumour characteristics are summarised in Table 2. The radiation dose and fraction used among all patients were 48 Gy in 15 fractions. Regarding the specific prescription dose, 11 patients received the prescription dose and the dose to PTV-boost in 4 patients was decreased to D_50%_ ≥ 48 Gy. The median age at diagnosis was 78 years (range 75–86 years), and nearly half of the patients were male. Eight (53.3%) and 7 (46.7%) patients showed PS of 0 and 1, respectively. According to the UICC Stage, all patients were in stage III. Primary tumours were located in the head/neck and the body/tail of the pancreas in 8 and 7 patients, respectively.

**Table 2:** Patient and tumour characteristics (n = 15)

Characteristics	No	%	Years
Age, median (range)			78 (75–86)
Sex			
Male	7	46.7	
Female	8	53.3	
PS			
0	8	53.3	
1	7	46.7	
TNM stage (UICC 8th)			
cT4N0M0 stage III	14	93.3	
cT4N1M0 stage III	1	6.7	
Tumour location			
Head/uncus	8	53.3	
Body/tail	7	46.7	

PS, performance status; UICC, Union for International Cancer Control; T, tumour; N, nodes; M, metastasis

### Treatment outcomes and recurrence pattern

**Fig. 1:**
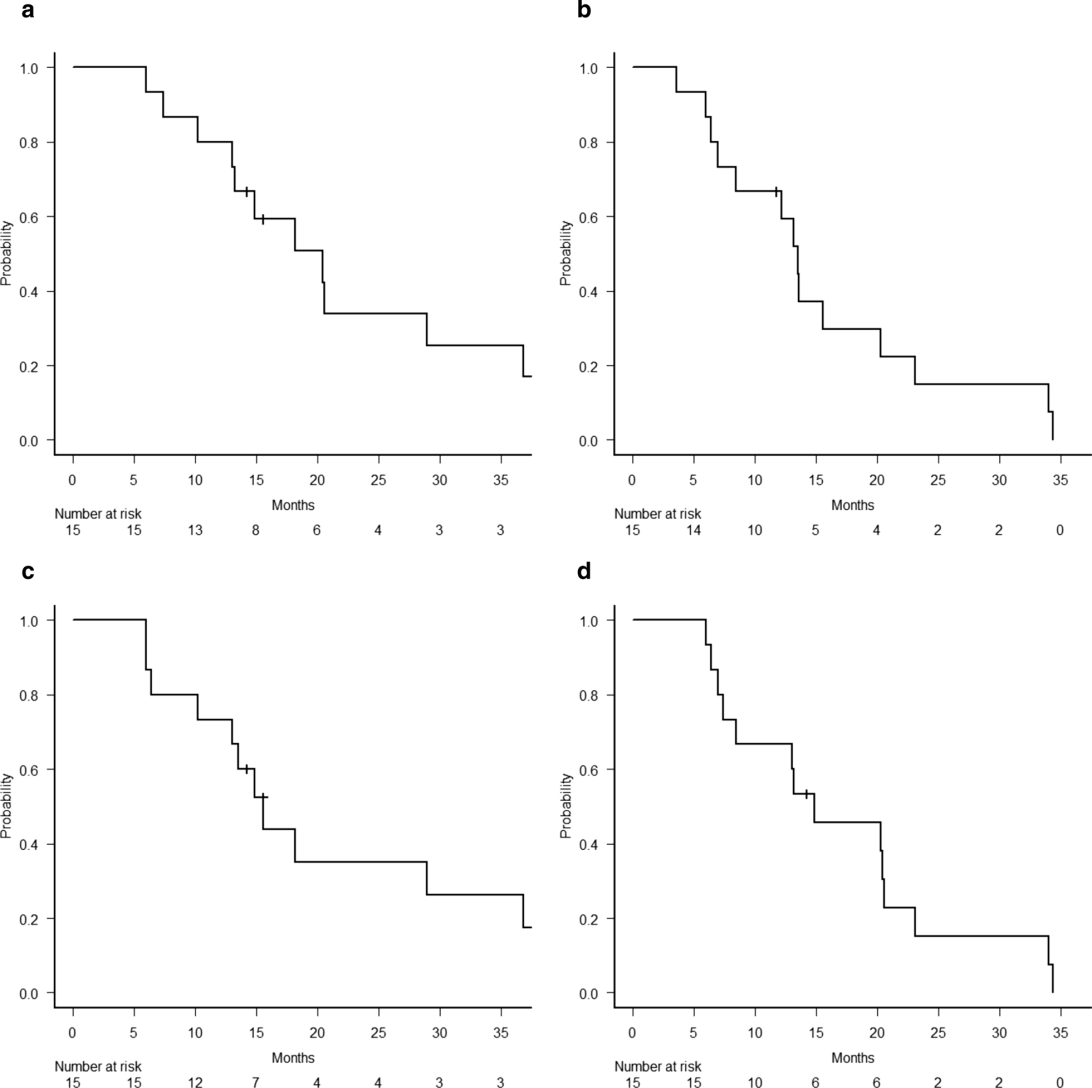
Kaplan–Meier estimates of **a** overall survival (OS), **b** progression-free survival (PFS), **c** locoregional progression-free survival (LRPFS), **d** distant metastasis-free survival (DMFS)

At the time of analysis, the median follow-up period was 15.5 months, and three patients (20%) were alive. The median survival time (MST) and median PFS were 20.4 [95% confidence interval (CI) 10.3–36.8] and 13.5 (95% CI 6.4–20.3) months, respectively. The 1-year OS and PFS rates were 80% (95% CI 50–93.1%) and 66.7% (95% CI 37.5–84.6%), respectively (Fig. 1). The median LRPFS and median DMFS were 15.6 (95% CI 6.4–36.8) and 14.9 (95% CI 7.0–20.5) months, respectively, whereas the 1-year LRPFS and DMFS rates were 73.3% (95% CI 43.6–89.1%) and 66.7% (95% CI 37.5–84.6%), respectively (Fig. 1). Among the 15 patients, 10 (66.7%) exhibited some recurrences during the follow-up period. Two patients (13.3%) had locoregional recurrence, six patients (40%) had distant metastasis, and two patients (13.3%) had both locoregional recurrence and distant metastasis when the first recurrence occurred. Peritoneal dissemination was observed in most cases with distant metastasis. The recurrence patterns are summarised in Table 3.

**Table 3:** Pattern of recurrence (n = 15)

Recurrence pattern	No. of patients (%)
Locoregional recurrence	2 (13.3)
Distant recurrence	6 (40)
Liver metastasis	2 (13.3)
Adrenal gland metastasis	1 (6.7)
Peritoneal dissemination	2 (13.3)
Multiple metastasis	1 (6.7)
Locoregional and distant recurrence	2 (13.3)
No evidence of recurrence	5 (33.3)

### Variation of chemotherapy

For induction CTX, 11 patients received gemcitabine alone, while four patients received gemcitabine in combination with nab-paclitaxel. The median period from the start of induction CTX to RT was 57 days (range 22–181 days). All patients received RT with concurrent gemcitabine. After RT, 14 patients underwent maintenance CTX except for one patient who had refused it. For maintenance CTX, 13 patients received gemcitabine and one patient received gemcitabine in combination with nab-paclitaxel until local recurrence or distant metastasis occurred. Because of worsened PS, adverse events, or patients’ requests, dose was reduced, or dose interval of gemcitabine was extended for induction, concurrent, and adjuvant CTX, respectively. The variation and reduction of CTX are summarised in Table 4.

**Table 4:** Variation and reduction of chemotherapy (CTX)

Induction CTX	No. of patients/total no. (%)	
	On schedule	Drug withdrawal
Gemcitabine 1000 mg/m^2^	5/15 (33.3)	4/15 (26.7)
Gemcitabine 800 mg/m^2^	1/15 (6.7)	1/15 (6.7)
Gemcitabine and nab-paclitaxel	0	4/15 (26.7)

Concurrent CTX	No. of patients/total no. (%)	
	On schedule	Drug withdrawal
Gemcitabine 1000 mg/m^2^	3/15 (20)	1/15 (6.7)
Gemcitabine 800 mg/m^2^	2/15 (13.3)	5/15 (33.3)
Gemcitabine 650 mg/m^2^	0	2/15 (13.3)
Gemcitabine 600 mg/m^2^	0	2/15 (13.3)

Adjuvant CTX	No. of patients/total no. (%)	
	On schedule	Drug withdrawal
Gemcitabine 1000 mg/m^2^	2/14 (14.2)	2/14 (14.2)
Gemcitabine 800 mg/m^2^	1/14 (7.1)	5/14 (35.7)
Gemcitabine 650 mg/m^2^	0	2/14 (14.2)
Gemcitabine 600 mg/m^2^	0	1/14 (7.1)
Gemcitabine and nab-paclitaxel	0	1/14 (7.1)

### Toxicities

Data of acute and late adverse events for all 15 patients are shown in Table 5. For haematologic toxicity during induction CTX, Grade 3 or higher anaemia, neutropaenia and thrombocytopaenia occurred in 1 (6.7%), 12 (80%), 1 (6.7%) patients, respectively. For acute toxicity, grade 3 or higher anaemia and neutropaenia were observed in 2 (13.3%), and 5 (33.3%) patients, respectively. Non-haematologic toxicity with grades 3 and 4 was observed during induction CTX and as acute toxicity. For late toxicity, Grade 3 diarrhoea and fatigue, arterial injury (pseudoaneurysm) were observed in 1 (6.7%) patient. Diarrhoea and fatigue occurred when patients were administered S-1 or UFT for recurrence. Any other grade 3 or higher late toxicities were not observed.

**Table 5:** Toxicities (n = 15)

	Grade 1	Grade 2	Grade 3	Grade 4
Toxicity during induction CTX				
Anaemia	2 (13.3%)	5 (33.3%)	1 (6.7%)	0
Neutropaenia	0	2 (13.3%)	9 (60%)	3 (20%)
Thrombocytopaenia	4 (26.7%)	5 (33.3%)	1 (6.7%)	0
Nausea	3 (20%)	0	0	0
Fatigue	8 (53.3%)	1 (6.7%)	0	0
Diarrhoea	1 (6.7%)	1 (6.7%)	0	0
Rash	3 (20%)	4 (26.7%)	0	0
Acute toxicity				
Anaemia	1 (6.7%)	6 (40%)	2 (13.3%)	0
Neutropaenia	0	7 (46.7%)	3 (20%)	2 (13.3%)
Thrombocytopaenia	5 (33.3%)	4 (26.7%)	0	0
Nausea	4 (26.7%)	1 (6.7%)	0	0
Fatigue	10 (66.7%)	1 (6.7%)	0	0
Diarrhoea	1 (6.7%)	2 (13.3%)	0	0
Rash	2 (13.3%)	0	0	0
Late toxicity				
Fatigue	4 (26.7%)	2 (13.3%)	1 (6.7%)	0
Diarrhoea	1 (6.7%)	3 (20%)	1 (6.7%)	0
Arterial injury^a^	0	0	1 (6.7%)	0
Duodenal ulcer	2 (13.3%)	0	0	0

^a^Means pseudoaneurysm

## Discussion

In elderly patients, aggressive treatment for malignant tumours is sometimes avoided because of age-associated functional decline, their severe comorbidities, or poor cognitive function [19]. This tendency is almost the same as that in pancreatic carcinoma [20–22]. In contrast, some studies reported that surgery or CTX could prolong OS for elderly patients with pancreatic carcinoma [21–23]. With regard to chemoradiotherapy for the elderly with LAPC, Miyamoto et al. suggested that the outcomes of patients treated with CCRT were similar to those of historical controls [24]. However, the patients in those studies were treated mainly with 5-FU and three-dimensional conformal radiotherapy (3D-CRT). While Rakhra et al. showed the benefit of hypofractionated CRT for LAPC and Francesca et al. assessed the efficacy of hypofractionated IMRT, no previous study focusing on elderly patients has been published [25, 26]. Therefore, our study evaluated hypofractionated CCRT using IMRT with high-dose gemcitabine for the elderly.

Based on several reports, the MST ranged from 8.6 to 16.6 months and the median PFS ranged from 6.0 to 12.0 months in LAPC patients treated with CCRT among all ages [2, 27–31]. Among elderly patients, Miyamoto et al. [24] demonstrated that the MST was 8.6 months in 24 LAPC patients treated with CCRT using 3D-CRT. Several previous CCRT studies for LAPC are summarised in Table 6. Our study showed that the MST and median PFS were 20.4 and 13.5 months, respectively, which were not inferior to the results of previous reports on CCRT. Kuroda et al. reported that CTX alone, which consisted of mostly GEM-based regimens for elderly pancreatic carcinoma patients, resulted in 9.0 months (274 days) of MST among 519 pancreatic carcinoma patients (approximately 28.3% of which were LAPC patients) [22]. However, it is difficult to compare our findings with that study because it included patients who had distant metastasis. Compared with these studies, our treatment resulted in favourable survival outcomes, in spite of the advanced age of our patients. Our previous study suggested that full dose gemcitabine and hypofractionated dose escalation with IMRT improved treatment outcomes, which could be applicable to elderly patients [8]. In addition, some recent studies demonstrated that high-dose radiation was a predictive factor for prolonged OS of patients with LAPC [31, 32]. In this study, we used a hypofractionated dose of 48 Gy in 15 fractions, of which the biological equivalent dose is almost equal to the conventional standard treatment dose (50.4–54 Gy in fractions of 1.8 Gy), by calculating with an α/β value of 10. However, considering the short overall treatment time, 48 Gy in 15 fractions is more potent than the conventional standard treatment dose and could contribute to better local control, which could lead to favourable survival outcomes.

**Table 6:** Summary of past CCRT studies for LAPC

Study (ref.)	Phase	No. of patients	Median age (y)	Radiotherapy technique	Radiotherapy dose (Gy)	Concurrent chemotherapy	MST (mo)	Median PFS (mo)	Local relapse rate (%)	1-year LRPFS (%)	Distant metastasis rate (%)	1-year DMFS (%)
Hammel et al. [2]												
Chemotherapy alone	III	136	63	–	–	–	16.5	8.4	46	N/A	44	N/A
CRT		133	5	3D-CRT	54	Cap 800 mg/m^2^	15.2	9.9	32		60	
Loehrer et al. [27]												
GEM alone	III	37	69	–	–	–	9.2	6.7	29.7	N/A	N/A	N/A
CRT		34	66	3D-CRT	50.4	GEM 600 mg/m^2^	11.1	6	11.7			
Chauffert et al. [29]												
GEM alone	III	60	62	–	–	–	13	N/A	N/A	N/A	N/A	N/A
CRT		59	60	3D-CRT	60	5-FU/CDDP	8.6					
Mukherjee et al. [28]												
GEM + RT	II	38	66	3D-CRT	50.4	GEM 300 mg/m^2^	13.4	10.4	55	N/A	68	N/A
Cap + RT		34	63.1			Cap 830 mg/m^2^	15.2	12	47		47	
Shibuya et al. [30]												
	II	21	65	3D-CRT	54	GEM 250 mg/m^2^	16.6	12	29	N/A	48	N/A
Miyamoto et al. [24]												
	Retro	24	78	3D-CRT (91.7%)	51.3 ± 2.0	5-FU (83.3%), 5-FU/GEM (12.5%)	8.6	N/A	20.8	N/A	66.7	N/A
Goto et al. [8]												
3D-CRT arm	Retro	80	65	3D-CRT	48.6–55.8	GEM 300 mg/m^2^	17.5	N/A	N/A	63.2	N/A	48.4
IMRT arm		27	66	IMRT	39–51	GEM 1000 mg/m^2^				73.1		49.3
Krishnan et al. [31]												
BED > 70 Gy	Retro	47	64	IMRT (96%)	83.11 (BED)	Cap (79%), GEM (16%)	17.8	8.6	N/A	21	N/A	N/A
BED ≤ 70 Gy		153	64	3D-CRT (91%)	59.47 (BED)	Cap (88%), GEM (11%)	15	5.3		9		
Current study												
	Retro	15	78	IMRT	48	GEM 1000 mg/m^2^	20.4	13.5	26.7	73.3	53.3	66.7

CRT, chemoradiotherapy; GEM, gemcitabine; RT, radiotherapy; Cap, capecitabine; 3D-CRT, three-dimensional conformal radiotherapy; IMRT, intensity-modulated radiotherapy; BED, biological equivalent dose; MST, median survival time; PFS, progression free survival; LRPFS, locoregional progression free survival; DMFS, distant metastasis free survival

As mentioned above, the OS and PFS were good; moreover, toxicity was also acceptable. Except one patient who had a pseudoaneurysm, grade 3 or higher non-haematologic adverse events induced by RT were not observed, and grade 3 non-hematologic toxicities because of CTX occurred in only two patients despite all patients being ≥ 75 years (Table 5). The reason why our treatment strategy was less toxic is as follows: First, the dose to the surrounding normal organs was reduced with IMRT, which was demonstrated in our previous study [8]. Second, in induction CTX, the dose and the interval of concurrent or maintenance CTX could be adjusted. As seen in Table 3, many of the patients needed a reduction in their CTX dose or an extension of their CTX interval because of adverse events or their PS. In fact, haematologic side effects seemed less toxic during CCRT than induction CTX, especially in neutropaenia (Table 5). The major aim of induction CTX in our protocol was to select patients who have adequate tolerability of CCRT and secure the time for planning IMRT [33]. Furthermore, as described by some previous studies, using the adjusted dose of CTX in reference to the dose of induction CTX could contribute to an increase in the completion rate of CCRT [28, 34]. As shown above, we adopted induction CTX before our CCRT using the IMRT protocol.

According to Table 3, the recurrence pattern was mainly distant metastasis, which was observed among 8 of 15 patients, whereas locoregional recurrence was observed among 4 of 15 patients. This trend was also reported in several previous studies [2, 35]. In the LAP07 study, OS was not prolonged compared to CTX alone, in spite of improving local control treated with CRT. The reason why local control could not improve OS may be because of the existence of occult metastasis for LAPC patients at the time of initial diagnosis; hence, systematic treatment is required for LAPC [1]. Conversely, local treatment could prolong OS among patients with no occult metastasis. Besides, local recurrence can cause obstructive cholangitis, duodenal obstruction, bleeding, or cancer pain. Therefore, improving local control by adding RT to CTX should be meaningful among LAPC patients, if this treatment could be tolerable. Since toxicity was not severe as we mentioned above, additional hypofractionated RT using IMRT may be good choice for elderly LAPC patients.

As this study was a retrospective and a single-arm analysis, there are several limitations. First, this analysis did not directly compare the results between CCRT and CTX alone or between IMRT and 3D-CRT for LAPC patients. Furthermore, we could not perform univariate or multivariate analyses for the prognostic factors because our sample size is small. A large number of randomised control trials are desirable to explore the most feasible therapy for elderly patients with LAPC. Second, our assessment of toxicities could be inaccurate. In particular, low-grade toxicity may have been underestimated because of the incomplete records on side effects, considering that this was a retrospective analysis. Nevertheless, we did not undercount severe toxicity, which should obviously be recorded, as it required additional medical care. Third, the PS of all patients in this study were 0 or 1 because these patients have been selected by our tumour board, and this result does not apply to all elderly patients. In other words, this treatment may be suitable for elderly patients with good PS who could tolerate chemoradiotherapy.

To the best of our knowledge, this is the first study to evaluate the outcomes and tolerability of hypofractionated CCRT using IMRT for elderly patients with LAPC. The results were favourable, and considering that the target was elderly people, hypofractionation may be meaningful because of its short treatment period. Since a long period of hospitalisation could induce disability such as cognitive decline or dementia in the elderly, short treatment is preferable. As the incidence of elderly patients with pancreatic carcinoma will increase, we should further investigate ideal treatments for LAPC patients, including other modalities.

## Conclusions

In summary, hypofractionated IMRT with concurrent gemcitabine in elderly LAPC patients with good ECOG PS resulted in favourable local control and survival outcomes without severe frequent toxicity. This treatment strategy may be one of the appropriate treatment options for elderly patients with good PS.

## Data Availability

The datasets used and analysed for this study and provided upon reasonable request.

## Abbreviations

IMRT: Intensity-modulated radiotherapy
LAPC: Locally advanced pancreatic carcinoma
OS: Overall survival
PFS: Progression-free survival
LRPFS: Locoregional progression-free survival
DMFS: Distant metastasis-free survival
ECOG: Eastern Cooperative Oncology Group
PS: Performance status
MST: Median survival time
CI: Confidence interval
CTX: Chemotherapy
CCRT: Concurrent chemoradiotherapy
RT: Radiotherapy
GI: Gastrointestinal
OARs: Organ at risks
UICC: Union for International Cancer Control
CT: Computed tomography
MRI: Magnetic resonance imaging
Gd-EOB-DTPA: Gadolinium-ethoxybenzyl-diethylenetriamine pentaacetic acid
FDG-PET: ^18^F-flurodeoxyglucose-positron emission tomography
CTCAE: Common Terminology Criteria for Adverse Events
GTV: Gross Tumour volume
CTV: Clinical target volume
PTV: Planning target volume
SIB: Simultaneous integrated boost
3D-CRT: Three-dimensional conformal radiotherapy
